# Revealing Metabolic Perturbation Following Heavy Methamphetamine Abuse by Human Hair Metabolomics and Network Analysis

**DOI:** 10.3390/ijms21176041

**Published:** 2020-08-21

**Authors:** Suji Kim, Won-Jun Jang, Hyerim Yu, Jihyun Kim, Sang-Ki Lee, Chul-Ho Jeong, Sooyeun Lee

**Affiliations:** 1 College of Pharmacy, Keimyung University, 1095 Dalgubeoldaero, Dalseo-gu, Daegu 42601, Korea; kimsuji921@naver.com (S.K.); mrdoin76@gmail.com (W.-J.J.); yhr2578@naver.com (H.Y.); 2 National Forensic Service, 10, Ipchun-ro, Wonju, Gangwon-do 26460, Korea; gadia95@korea.kr (J.K.); skleedoc@korea.kr (S.-K.L.)

**Keywords:** methamphetamine, drug addiction, metabolomics, network analysis, hair, lipid metabolism

## Abstract

Methamphetamine (MA) is a highly addictive central nervous system stimulant. Drug addiction is not a static condition but rather a chronically relapsing disorder. Hair is a valuable and stable specimen for chronic toxicological monitoring as it retains toxicants and metabolites. The primary focus of this study was to discover the metabolic effects encompassing diverse pathological symptoms of MA addiction. Therefore, metabolic alterations were investigated in human hair following heavy MA abuse using both targeted and untargeted mass spectrometry and through integrated network analysis. The statistical analyses (*t*-test, variable importance on projection score, and receiver-operator characteristic curve) demonstrated that 32 metabolites (in targeted metabolomics) as well as 417 and 224 ion features (in positive and negative ionization modes of untargeted metabolomics, respectively) were critically dysregulated. The network analysis showed that the biosynthesis or metabolism of lipids, such as glycosphingolipids, sphingolipids, glycerophospholipids, and ether lipids, as well as the metabolism of amino acids (glycine, serine and threonine; cysteine and methionine) is affected by heavy MA abuse. These findings reveal crucial metabolic effects caused by MA addiction, with emphasis on the value of human hair as a diagnostic specimen for determining drug addiction, and will aid in identifying robust diagnostic markers and therapeutic targets.

## 1. Introduction

Methamphetamine (MA) is a highly addictive central nervous system stimulant whose abuse is a critical public health issue because of the acute complications associated therewith, particularly hyperthermia, long-term neurotoxicity, and strong potential for dependence [1,2]. MA remains a widely used illicit drug in the US according to the 2016 National Survey of Drug Use and Health, which reported that 6.5% of people aged 26 years and older have used MA [3]. It is also the most problematic drug of abuse showing an increasing trend among youths in East Asian countries, such as South Korea, Japan, and China [4].

Drug addiction is not a static condition but a chronically relapsing disorder, which develops from the repetition of positive and negative effects caused by temporary drug use and withdrawal, respectively. In this cycle, the brain reward system is highly activated during the transition to drug dependence and less activated at the addiction stage. During abstinence, it almost returns to normal [5,6]. Therefore, clinical laboratory criteria for the diagnosis and treatment of drug addiction are not fully elucidated and no specific biomarkers are practically available.

Metabolomics is a powerful tool for the discovery of potential diagnostic, prognostic, and therapeutic biomarkers for diseases, including drug addiction [7,8]. Based on the theory proposed from the development of methadone maintenance programs, addiction could be considered a metabolic disease as it initiates from a disruption in metabolism and causes persistent neurochemical disturbances leading to addiction [8,9]. Several metabolomic studies in animals were recently conducted to investigate MA use and revealed that MA exposure caused an imbalance in metabolites related to energy metabolism, amino acid metabolism, phospholipids metabolism, oxidative stress, and neurotransmitters [10,11,12,13,14,15,16] However, the altered metabolites or metabolic pathways were inconsistent, depending on the doses and durations of MA administration and targeted animal model samples [17]. Moreover, knowledge gained from the metabolic analysis is often fragmentary owing to the bottleneck in identifying metabolites in case of untargeted metabolomics and the lack of further in-depth study based on the biological importance of metabolic alterations caused by MA. In particular, additional bioinformatic analyses for interpreting metabolomics data have been performed to a lesser extent than those for interpreting other -omics data. Metabolic pathway-related network analysis could facilitate in comprehensively understanding the biological functions of metabolic alterations, and the data obtained could prove useful in using the identified metabolites or metabolic pathways as potential markers for MA addiction.

Metabolic changes in biofluids, such as blood or urine, collected at specific time points are highly dynamic. Thus, clarifying conclusive metabolic disturbances in drug addiction caused by repetitions of the drug dependence cycle using blood or urine analysis is not straightforward. Recently, hair metabolomics was suggested as a more appropriate tool than conventional ones for the discovery of robust biomarkers, in particular for chronic diseases [18,19] and pregnancy complications [20,21,22] or embryopathy [23] via maternal hair metabolome profiling. Hair is a valuable specimen reflecting the effects of chronic toxicant exposure because of its extended window of detection, stable and repeatable measurement of accumulated substances, and easy sample storage [24]. Furthermore, a previous study on the comparison of the expression of messenger RNA in postmortem human brains and scalp hair follicles showed that scalp hair follicles could be a beneficial genetic biomarker source for brain diseases [25].

For the reliable diagnosis and efficient treatment of MA addiction, the understanding of decisive metabolic perturbations and the discovery of robust biomarkers that are applicable even during abstinences are necessary. Experimental studies or clinical reports on the effect of drug addiction on metabolic changes are lacking. In particular, few metabolic studies on human hair in drug addiction have been conducted. In the present study, metabolic alterations following heavy MA abuse were investigated via human hair metabolomic analysis using both targeted and untargeted mass spectrometry (MS) followed by integrative network analysis.

## 2. Experimental Section

### 2.1. Materials and Chemicals

Formic acid, 2-aminoantracene, and 2,3,4,5,6-pentafluorobenzoic acid were purchased from Sigma-Aldrich (St. Louis, MO, USA). AbsoluteIDQ p180 kit for targeted analysis was purchased from Biocrates Life Sciences AG (Innsbruck, Austria). All solvents were of high-performance liquid chromatography (HPLC) grade. All other chemicals were of reagent grade.

### 2.2. Subjects

Hair samples from heavy MA abusers (males with black hair; *n* = 10; Table 1) were selected from those submitted to the National Forensic Service in Seoul, Korea, by the police for the purpose of illegal drug abuse detection between 2017 and 2018 (total number of cases = 15,415). The police investigation informed of only MA use, and not of any other drugs such as cannabis, cocaine, heroin, and other new psychoactive drugs for the cases selected for this study. The drug consumption of the subjects was confirmed by both routine urine testing (immunoassay as a preliminary test, followed by HPLC-MS/MS as a confirmation test) and hair testing (HPLC-MS/MS) for amphetamines, 11-nor-9-carboxy-Δ9-tetrahydrocannabinol, cocaine, opiates, and benzodiazepines in the laboratory. In the present study, only cases that were positive in both the urine and hair samples for MA and its major metabolite, (AM), were chosen. The chronic heavy use of cigarettes and alcohol was excluded, based on the police investigative report.

The severity of chronic MA consumption was evaluated based on the drug and/or metabolite concentrations in the hair. The concentrations of MA and AM in the hair were determined using a fully validated method reported previously [26]. Statistical evaluations, where the low, medium, and high ranges for hair drug concentrations were defined using the minimum to the 25th percentile, 25th to 75th percentile, and 75th percentile to the maximum, respectively, were performed based on previous studies to identify heavy heroin [27], cocaine [28], and MA [29] drug users. Subjects were considered heavy MA abusers if MA concentrations in hair strands measuring 3 cm from the root were >24.5 ng/mg [29].

The hair samples from drug-free controls (males, *n* = 12, black hair, Table 1) were collected based on the answers of the questions of the Korean version of the National Institution of Drug Abuse modified Alcohol, Smoking and Substance Involvement Screening Test [30]. The chronic heavy use of cigarette and alcohol as well as the use of other drugs was excluded. The study was approved by the Institutional Review Board of Bugok National Hospital (Gyeongsangnam-do, Republic of Korea, approval number: BNH-2018-03, approval date: 26 April 2018).

### 2.3. Targeted Metabolomics

An AbsoluteIDQ p180 kit, which allows the simultaneous quantification of 187 metabolites (40 acylcarnitines, 42 amino acids and biogenic amines, 90 glycerophospholipids, and 15 sphingolipids) and a sum of hexoses including glucose, was used for hair sample preparation. Hair strands measuring 3 cm from the root were washed, dried, and finely cut. Hair segments of approximately 20 mg were incubated for 16 h in 2 mL methanol. The extract was evaporated to dryness and the residue was reconstituted in 100 μL of methanol. Ten microliters of the methanol extract, with 10 μL of the solution of internal standards, composed of 46 isotope-labeled and chemically homologous internal standards, was loaded into the kit, derivatized, and extracted according to the manufacturer’s instructions [31]. After filtering, the filtrate was subjected to both flow injection analysis (FIA)-MS/MS and HPLC-MS/MS.

An AB Sciex 4000 QTrap mass spectrometer (Sciex, Framingham, MA, USA) was used for analysis in the multiple reaction monitoring detection mode with electrospray ionization (ESI). Forty microliters of the prepared samples were subjected to MS/MS by FIA for the determination of amino acids and biogenic amines present, and 10 μL of these samples was introduced by HPLC to MS/MS for the determination of other groups. For the FIA-MS/MS analysis, the sample was run with the solvent provided by the manufacturer at the following rates: 0–1.6 min, 0.03 mL/min; 1.6–2.4 min, 0.2 mL/min; 2.4–2.8 min, 0.2 mL/min; and 2.8–3.0 min, 0.03 mL/min. For the HPLC-MS/MS analysis, a Zorbax Eclipse XDB C18 column (3.0 mm × 100 mm, 3.5 μm; Agilent) was used. The mobile phase comprised 0.2% formic acid in water (A) and 0.2% formic acid in acetonitrile (B), and the gradient conditions were as follows: 0–0.5 min, 0% (B); 0.5–5.5 min, 0–95% (B); 5.5–6.5 min, 95% (B); 6.5–7 min, 95–0% (B); and 7–9.5 min, 0% (B). The flow rate was 0.5 mL/min. The kit was validated using MetValTM (Biocrates Life Sciences AG) software, and the analytical results were processed using AnalystTM (Sciex) and MetValTM software.

### 2.4. Untargeted Metabolomics

Ultra-high-performance liquid chromatography coupled to quadrupole time-of-flight electrospray ionization mass spectrometry (UPLC-QTOF-ESI-MS) analyses for hair samples were performed as previously reported [18] with minor modifications. The hair extract prepared according to the method described above was evaporated to dryness and the residue was reconstituted in 100 μL of a solution of methanol and 0.1% formic acid in water (9:1) before filtering through a 0.45-μm polyvinylidene fluoride microporous membrane. Before methanol incubation, 2-aminoanthracene (100 μg/mL, 50 µL) and 2,3,4,5,6-pentafluorobenzoic acid (1 mg/mL, 5 μL), used as internal standards for positive and negative ESI modes, respectively, were added. Finally, 5 μL was injected into the LC-QTOF-ESI-MS system (Agilent 6530 Accurate-Mass Q-TOF LC/MS System with Agilent 1290 Infinity LC, Agilent Technologies, Palo Alto, CA, USA). Pooled quality control (QC) samples, in which a 5 μL aliquot of each sample was mixed together, were analyzed along with authentic hair samples.

The guard column, Zorbax SB-C8 (3.5 μm, 2.1 × 30 mm, Agilent Technologies) and the analytical column, Zorbax SB-Aq (1.8 μm, 2.1 × 100 mm, Agilent Technologies) were maintained at 40 °C. The mobile phase consisted of 0.1% formic acid in water (A) and 0.1% formic acid in acetonitrile (B). The gradient conditions were as follows: 0–30 min, 1–20% B; 30–40 min, 20–90% B; 40–45 min, 90% B; 45–47 min, 90–1% B; 47–52 min, 1% B, at a flow rate of 400 μL/min.

The MS system was operated using ESI in the positive and the negative ionization modes. The optimized MS system conditions for both ionization modes were as follows: drying gas temperature, 300 °C; drying gas flow, 10 L/min; nebulization pressure, 45 psi; sheath gas temperature, 350 °C; sheath gas flow, 10 L/min; capillary voltage, 3500 V; nozzle voltage, 0 V; fragmentor voltage, 175 V; and skimmer voltage, 65 V. The mass range was 50–1700 *m*/*z* and the scan rate was 2.00 spectra/s. Purine (exact mass for [M + H]^+^ = 121.050873) and 1,2,3,4,5,6-hexakis(2,2,3,3-tetrafluoropropoxy)-1,3,5,2,4,6-triazatriphosphinane (exact mass for [M + H]^+^ = 922.009798) were used for mass calibration.

### 2.5. Data Processing and Statistical Analysis

In targeted metabolomics, data cleaning was performed with reference to the criteria used in previous studies [32,33]. Values greater than the limit of detection (LOD) in the raw data of each group were chosen for (semi-) quantification. To calculate fold-changes, values below LOD in each group were replaced with LOD/2 or random values between 0 and LOD.

Data processing was performed using MassHunter Profinder (version B.06.00) and Mass Profiler Professional (MPP, version B.13.1) software (Agilent Technologies) in untargeted metabolomics. With regard to UPLC-QTOF-ESI-MS data, potential molecular features were first extracted using the batch recursive feature extraction algorithm in Profinder using MS raw data. Parameters for feature extraction were as follows: peak height >400 counts; ion species, [M + H]^+^ and [M + Na]^+^ for positive ions, and [M − H]^−^ for negative ions; isotope peak spacing tolerance, 0.0025 *m*/*z* and 7.0 ppm; and charge state ≤1. Features were aligned at a retention time window of 0.50 min and a mass window of 10 ppm + 2 mDa. All extracted peaks based on particular masses and retention times were inspected to reduce both false negative and false positive features. The assignment of metabolites to ion features was performed using the in-house database [18].

The online MetaboAnalyst 4.0 software (http://www.metaboanalyst.ca/MetaboAnalyst/faces/home.xhtml) was used for statistically analyzing the data obtaining from both untargeted and targeted analyses. The data were normalized by the median, and the unpaired *t*-test was used to determine significant differences in metabolites or ion features. The following criteria were applied: 80% frequency and fold-change >1.2 with *p*-values <0.05 between groups. The metabolic differences between groups were evaluated using principal component analysis (PCA). The analytical data were modeled using partial least squares-discriminant analysis (PLS-DA) to reveal the differences between the two groups, and the metabolites or ion features contributing to the separation were selected as the variable importance on projection (VIP) value. Five-component and 10-fold cross-validation algorithms were used to prevent the over-fitting of the PLS-DA model. R2 and Q2 parameters were used to evaluate the model, mean fitness, and prediction ability. Receiver-operator characteristic (ROC) curve analysis was performed to investigate the potential of metabolites and ion features as biomarker candidates.

Network analysis was performed using a web-based analysis tool, Prize-collecting Steiner forest algorithm, for the integrative analysis of untargeted metabolomics (PIUMet, http://fraenkel-nsf.csbi.mit.edu/piumet2/). The parameters for the network analysis were as follows: w (a parameter for tuning the number of trees in the resulting network), 10; mu (a parameter for tuning the number of input nodes included in the output), 0.0005; beta (a parameter for controlling the bias toward high-degree nodes), 2.0; R (a parameter for running PIUMet for R times by adding random noise to the interactome and calculating robustness scores), 1. In addition, metabolic pathway analysis was performed using Cytoscape software (v3.7.1, https://cytoscape.org/) according to the Kyoto Encyclopedia of Genes and Genomes (KEGG, Release 93.0, 1 January 2020) pathway database.

## 3. Results

### 3.1. Targeted Metabolomics

**Figure 1:**
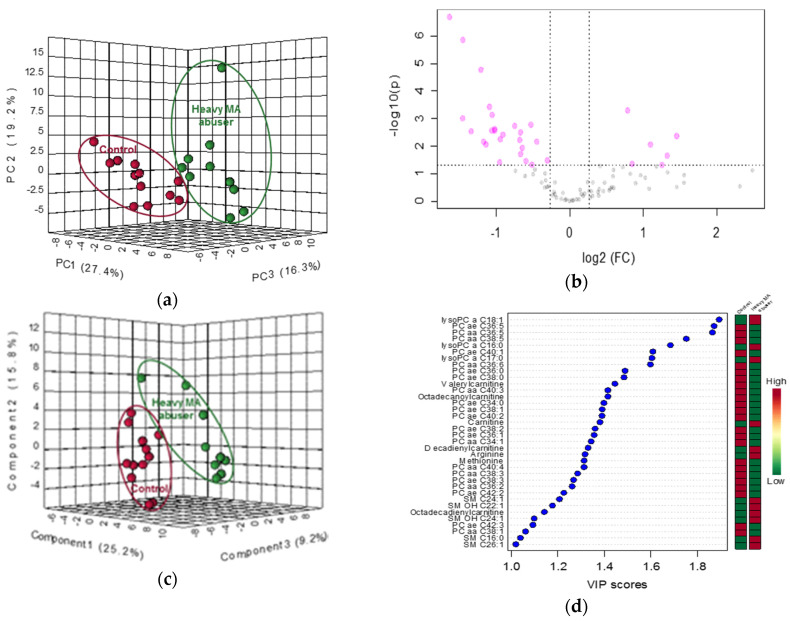

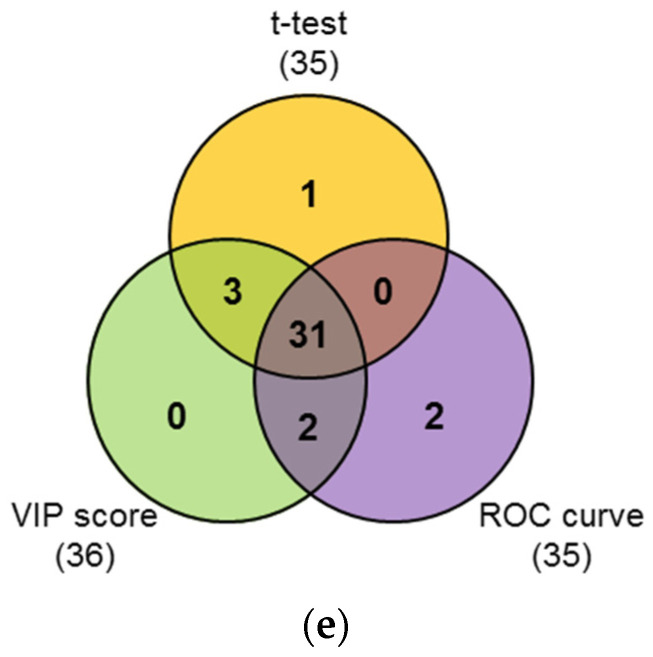
Statistical results of targeted metabolomics in hair samples from drug-free controls (*n* = 12) and heavy MA abusers (*n* = 10). (**a**) Principal component analysis (PCA) score plot. Red circle: drug-free controls; green circle: heavy MA abusers. (**b**) Volcano plot; red circle: metabolites with significance at *p* < 0.05 and fold change > 1.2 or < −1.2. (**c**) Partial least squares discriminant analysis (PLS-DA) score plot. Red circle: drug-free controls; green circle: heavy MA abusers. (**d**) Variable importance on projection (VIP) score plot. Colored box: relative concentration in drug-free controls and heavy MA abusers; (**e**) Venn diagram integrating results from the *t*-test, VIP scores, and receiver-operator characteristic (ROC) curves.

**Figure 2:**
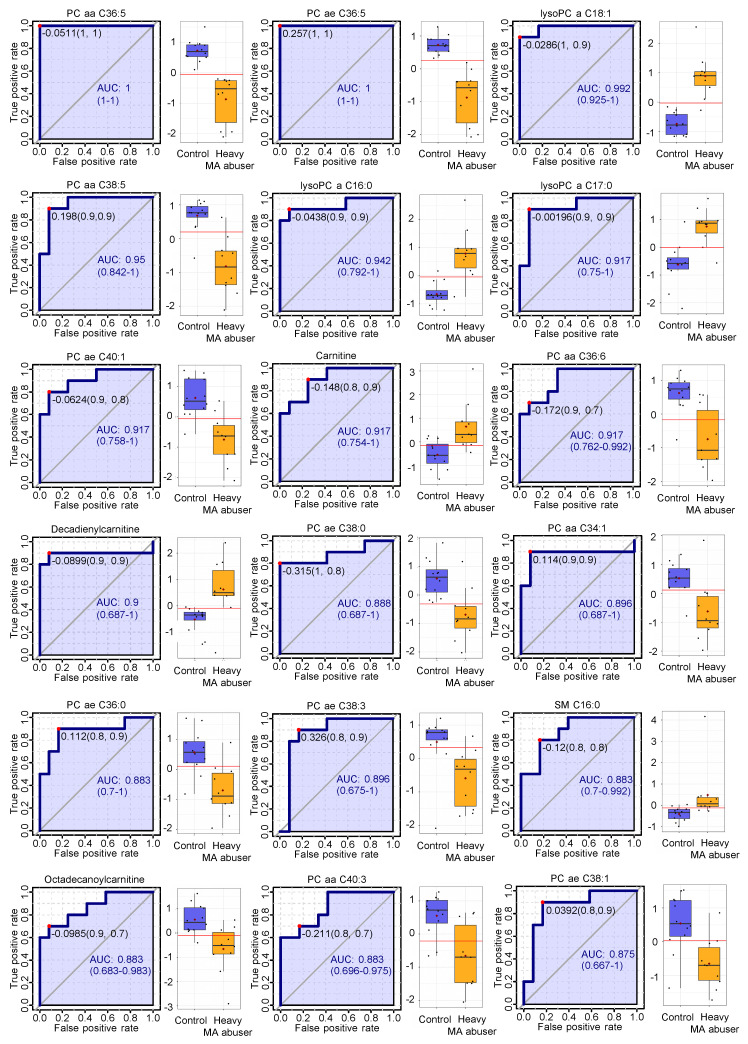

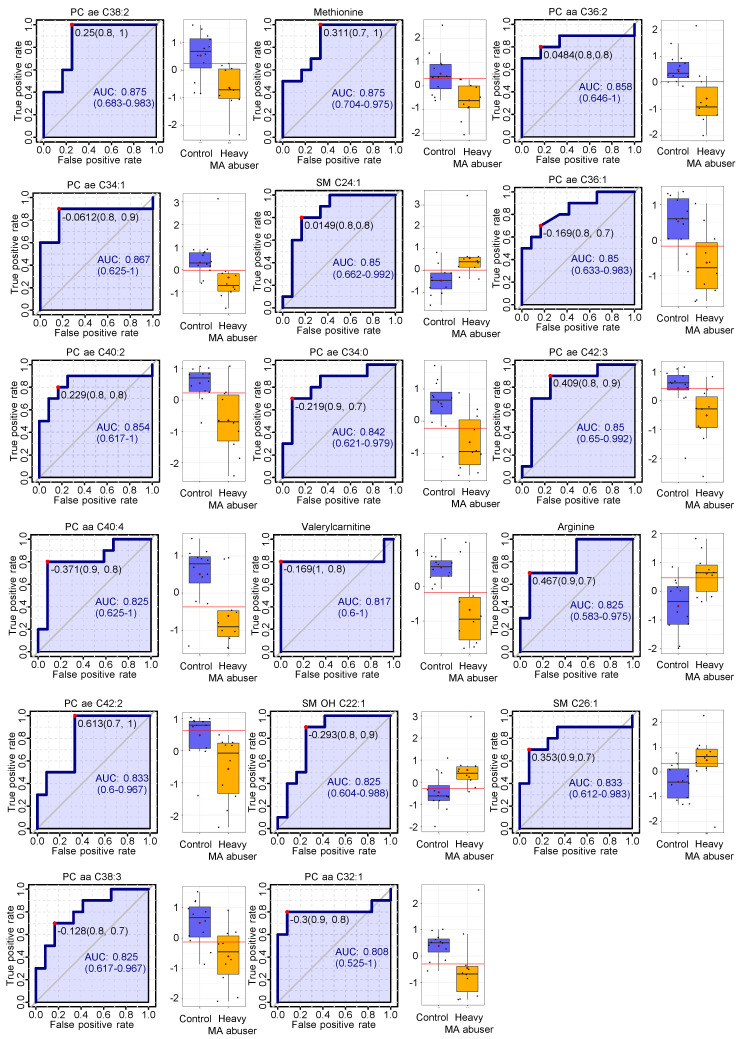
Results of the receiver operating characteristic (ROC) curve analysis of the 37 metabolites from *t*-test, variable importance on projection scores, and ROC curves in targeted metabolomics and metabolite levels. AUC: area under the ROC curve.

Targeted metabolomics analysis was conducted using a commercial analytical tool for the simultaneous quantification of 188 metabolites; of these, 90 (22 acylcarnitines, 22 amino acids and biogenic amines, 39 glycerophospholipids, and 7 sphingolipids) were (semi-) quantified following data cleaning (Table 2). The PCA score plot derived from the targeted metabolomics data indicated that the drug-free control group and heavy-MA abuse group were separated and described by 24.7% of the first principal component (PC1) (Figure 1a). Among the 90 metabolites, 11 and 24 metabolites were significantly up and down-regulated, respectively, following heavy MA abuse (fold-change > 1.2, *p* < 0.05, Figure 1b, Table 3). To further identify the metabolites that resulted in significant differences between the two groups, PLS-DA was performed. Four components were used to construct the model with R2 and Q2 values of 0.983 and 0.885, respectively. Results revealed that the two groups were clearly separated (Figure 1c). Metabolites with VIP values greater than 1.0 were considered significant, and 36 metabolites, including 24 glycerophospholipids, were identified (Figure 1d). The results of the ROC curve analysis demonstrated that the areas under the curve of the 35 metabolites were greater than 0.8. Thirty-one metabolites were commonly critical as identified by *t*-test, VIP scores, and ROC curve (Figure 1e). The results of the ROC analysis and the level of the aforementioned 35 critically altered metabolites are shown in Figure 2. The significance test demonstrated that valerylcarnitine and octadecanoylcarnitine were significantly downregulated, whereas carnitine and decadienylcarnitine were significantly up-regulated in the acylcarnitine group. The level of arginine was significantly increased whereas that of methionine was decreased in the hair samples from the heavy MA abusers. Most metabolites (semi-)quantified in the current study were lipids. Among the levels of 39 glycerophospholipids, those of the four lysophosphatidylcholines (lysoPCs) were increased and those of the 21 PCs were decreased following heavy MA abuse. Among the levels of seven sphingolipids, those of the three sphingomyelin (SM) increased (Table 3).

### 3.2. Untargeted Metabolomics

**Figure 3:**
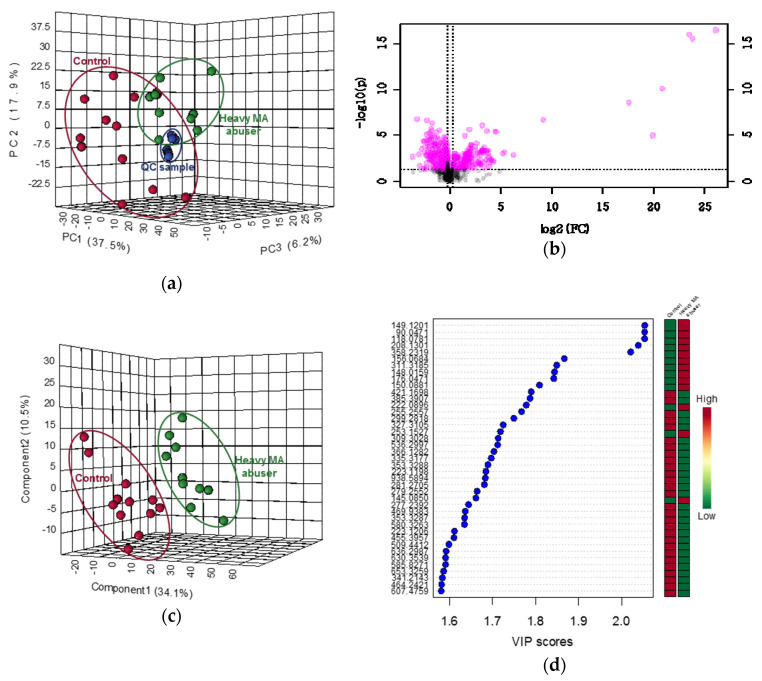

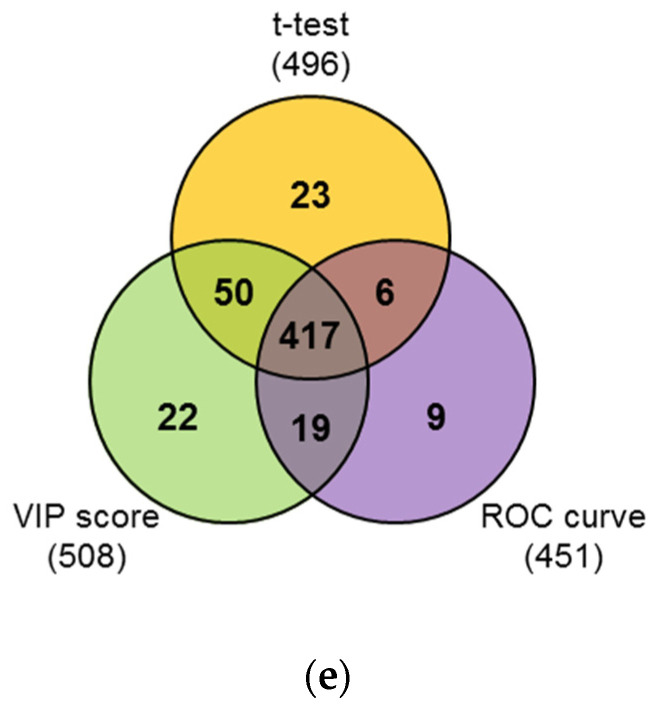
Statistical results of untargeted metabolomics by the positive ionization mode of ultra-high-performance liquid chromatography coupled to quadrupole time-of-flight electrospray ionization mass spectrometry (UPLC-QTOF-ESI-MS) in hair samples from drug-free controls (*n* = 12) and heavy MA abusers (*n* = 10). (**a**) Principal component analysis (PCA) score plot. Red circle: drug-free controls; green circle: heavy MA abusers. (**b**) Volcano plot. Red circle: metabolites with significance at *p* < 0.05 and fold change > 1.2 or < −1.2: +ND: increased from the value of not detected. (**c**) Partial least squares discriminant analysis (PLS-DA) score plot. Red circle: drug-free controls; green circle: heavy MA abusers. (**d**) Variable importance on projection (VIP) score plot. Colored box: relative concentration in drug-free controls and heavy MA abusers; (**e**) Venn diagram integrating results from the *t*-test, VIP scores, and receiver-operator characteristic (ROC) curve.

**Figure 4:**
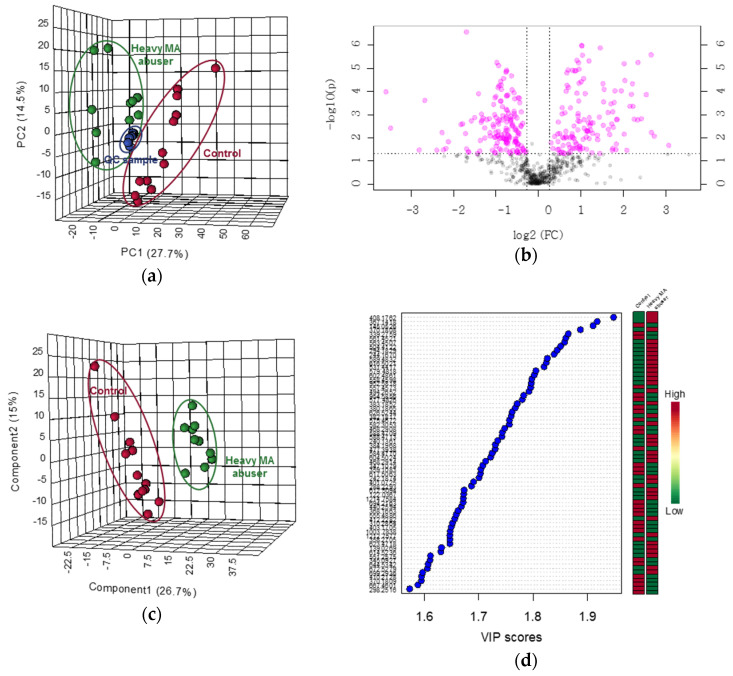

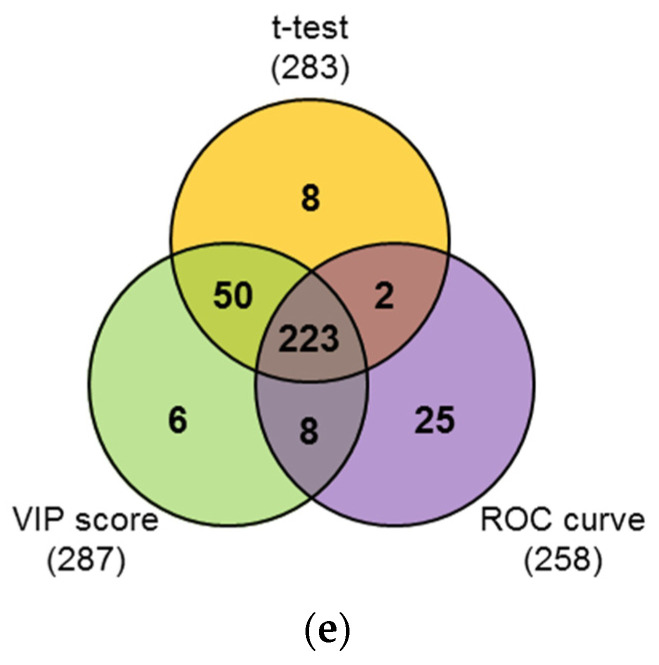
Statistical results of untargeted metabolomics using the negative ionization mode of quadrupole time-of-flight electrospray ionization mass spectrometry (UPLC-QTOF-ESI-MS) in hair samples from drug-free controls (*n* = 12) and heavy MA abusers (*n* = 10). (**a**) Principal component analysis (PCA) score plot. Red circle: drug-free controls; green circle: heavy MA abusers. (**b**) Volcano plot. Red circle: metabolites with significance at *p* < 0.05 and fold change > 1.2 or < −1.2. (**c**) Partial least squares discriminant analysis (PLS-DA) score plot. Red circle: drug-free controls; green circle: heavy MA abusers. (**d**) Variable importance on projection (VIP) score plot. Colored box: relative concentrations in drug-free controls and heavy MA abusers. (**e**) Venn diagram integrating results from the *t*-test, VIP scores, and receiver-operator characteristic (ROC) curves.

The statistical results of untargeted metabolomics are shown in Figure 3 and Figure 4. The PCA score plots derived from all ion features produced by positive and negative ionization modes of UPLC-QTOF-ESI-MS are displayed in Figure 3a and Figure 4a. The drug-free control group and heavy-MA abuser group were distinguished and described by 34.7% and 28.3% of the PC1, respectively, and the QC samples were closely clustered, according to the ion features detected in each analytical mode. Among the 994 (positive ionization) and 677 (negative ionization) ion features, 496 and 283 were significantly up- or downregulated, respectively, following heavy MA abuse (fold-change > 1.2, *p* < 0.05, Figure 3b and Figure 4b). In the PLS-DA score plots, a remarkable difference in the two groups was observed. Five components were used to construct the models, with R2 and Q2 values of 0.995 and 0.772 in the positive ionization mode and of 0.997 and 0.803 in the negative ionization mode, respectively (Figure 3c and Figure 4c). On this basis, the variables differing between the groups were filtered using VIP values greater than 1.0, and 508 (positive ionization) and 287 (negative ionization) ion features were identified. The lists of ion features with the top 10% of the VIP values are detailed in Figure 3d and Figure 4d. Many of the ion features that satisfied the significant criteria were identified to be at the area under the curve of the ROC curve higher than 0.8 (Supplementary material 1). The number of metabolites that were commonly critical as identified using *t*-test, VIP score, and ROC curve were 417 and 223 for positive and negative ionization mode for each (Figure 3e and Figure 4e). Table 4 shows the metabolites analyzed using UPLC-QTOF-ESI-MS and identified using our in-house library, with retention times and mass spectra. Significance tests indicated that the levels of 4-guanidinobutanoate and 4-hydroxybenzaldehyde significantly increased, while those of sphinganine, urate, and petroselinic acid significantly decreased.

### 3.3. Network Analysis

**Figure 5:**
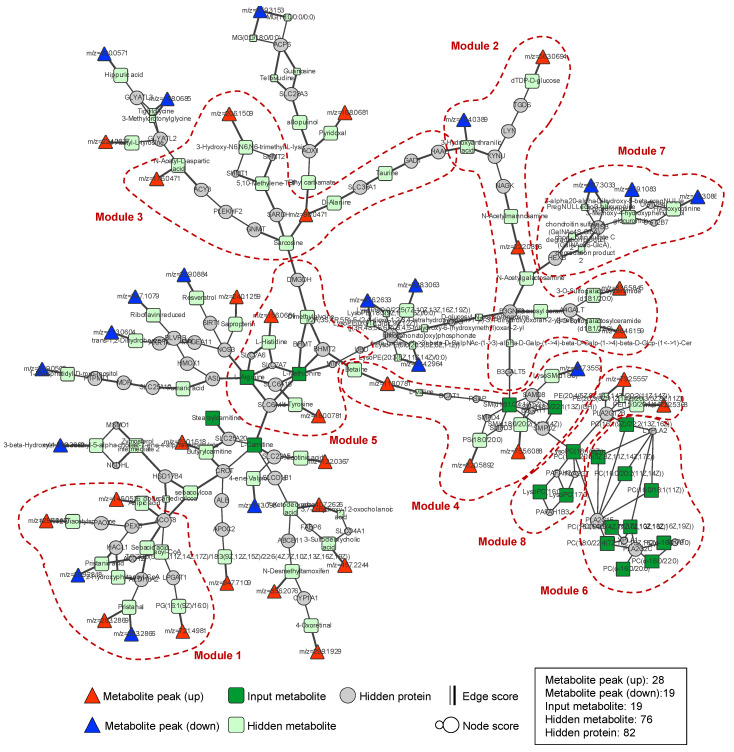
Altered metabolic pathways identified by PIUMet.

To understand the alteration of the metabolites found in our data, we performed integrated network analysis of critical metabolites (34) and ion features (417 and 223 for positive and negative ionization modes, respectively) from targeted and untargeted metabolomics, and the results of these are shown in Figure 5 and Table 5. The number of input terminals of the network was as follows: metabolites from targeted metabolomics, 21 and ion features from untargeted metabolomics, 198. The 198 ion features were matched with 664 metabolites in the Human Metabolome Database and Recon database (Supplementary material 2). Among these, 58 ion features were matched with 226 metabolites in the PIUMet database (Figure 5). The resulting network consisted of 224 nodes, 262 edges, 19 targeted metabolites, and 47 ion features. The biosynthesis or metabolism of lipids, such as glycosphingolipids, sphingolipids, glycerophospholipids, and ether lipids, as well as the metabolism of amino acids (glycine, serine and threonine; cysteine and methionine) was significantly affected by heavy MA abuse (*p* < 0.00001, Table 2). The node genes for each metabolic pathway are listed in Table 5 and Supplementary material 3.

## 4. Discussion

The main advantage of this study is the well-characterized human hair metabolic investigation method, using metabolomics combined with network analysis, through which detailed metabolic examination was achieved for heavy MA abuse. Human hair, as a metabolomics sample, enables us to better understand the metabolic consequences encompassing the diverse conditions of MA addiction. Only limited information is available regarding the effect of drug abuse or addiction on endogenous metabolites in the human hair. Xie et al. previously reported that sorbitol and cortisol were upregulated while arachidonic acid, glutathione, linoleic acid and myristic acid were downregulated, based on metabonomic study in heroin abusers’ hair using LC-ion trap-TOF MS [34]. However, the metabolite coverage was restricted to fully understand the perturbation of metabolism related to drug addiction. To the best of our knowledge, this is the first report to investigate metabolic alterations extensively in human hair from heavy drug abusers using metabolomics combined with network analysis.

The main limitation of this study is the relatively small sample size; however, this was compensated by the strict selection of samples only for MA use, by the results of qualitative urine drug testing and quantitative hair drug testing as well as the police investigative reports in a total of 15, 415 cases. The hair samples were all black proximal hair of 3 cm in length, to exclude the effect of the MA use period and melanin contents on the levels of hair metabolomes. The hair strands, measuring 3 cm from the root, represent a MA use history of three months prior to sampling, based on a rate of hair growth of approximately 1 cm/month [35]. Moreover, the previous study on the relationships between MA dose, frequency of use, and MA concentrations in hair [36] reported high correlations between the cumulative doses of MA use calculated from self-reported daily dose and frequency with the concentrations of MA (r = 0.87) and AP (r = 0.78). Based on the high MA concentrations in the hair determined, subjects in the present study were considered heavy MA abusers. Another limitation of this study, potentially shared by all untargeted metabolomics approaches, is the difficulty in identifying metabolites for numerous ion features. However, we were able to focus on the 19-targeted metabolites and 47-ion features included in the metabolic network to elucidate biological meaning through PIUMet. Currently, human metabolomics data concerning drug addiction are limited, compared with data for other metabolic diseases. Drug addiction-related potential biomarkers proposed in animal studies need to be validated in clinical settings. Therefore, our findings merit confirmation in larger human studies.

The drug-free control and heavy MA abuser groups were clearly separated by PCA and PLS-DA conducted on the data from the targeted and untargeted analyses. The authors of previous studies observed an inconsistent group separation in MA-induced conditioned place preference (2 mg/kg for 10-day conditioning) and acute MA intoxication (10 mg/kg/h for 4 h), based on metabolic changes observed in animal studies. The control and treated groups in the latter study were well separated based on their urine metabolomes while they were not in the former. The authors proposed that this may be attributed to the different actions on the brain reward circuitry and the resulting adaptation to chronic MA use [12,15]. In another previous study, metabolic alterations in urine and hair from self-administration of MA in rats were observed using untargeted metabolomics. Notably, large numbers of ion features (1484 and 526 in the positive and the negative ESI mode, respectively) were extracted in rat hair, with a significant difference between the hair samples collected before and after MA self-administration was observed in the PCA score plot. In addition, the levels of more ion features were significantly altered in hair (103 and 18 in the positive and the negative ionization mode of untargeted MS, respectively) than in urine after MA self-administration, suggesting that hair may be a more suitable diagnostic specimen for evaluation of drug addiction [18]. Limited clinical studies have been performed to study the effect of MA exposure by analyzing specific target metabolites, such as glutamate [37] and n-acetyl-aspartate [38], in the brain.

The lipophilicity and basicity of compounds are known to influence their incorporation into the hair from the bloodstream. Lipophilic and uncharged molecules can easily cross membranes and exist in matrix cells [35,39]. However, amino acids, organic acids, and fatty acids were most often identified in previous human hair metabolic studies [20,21,22,23]. In our study, 22 acylcarnitines, 22 amino acids and biogenic amines, 39 glycerophospholipids, and 7 sphingolipids were (semi-)quantified in human hair, and their distribution was investigated on the basis of heavy MA abuse in the targeted analysis.

Acylcarnitines belong to a group of markers for mitochondrial function, in particular, the β-oxidation of fatty acids. Elevated acylcarnitines cause lipotoxicity in the heart, liver, skeletal muscle, and brain, and induce mitochondrial dysfunction [40,41]. The alteration of the levels of some acylcarnitines, including carnitine, decadienylcarnitine, octadecadienylcarnitine, octadecanoylcarnitine, varerylcarnitine, in the current study may imply a dysregulation of β-oxidation following chronic MA abuse. However, few studies assessing the effects of addictive drugs on β-oxidation have been performed. A previous study reported accumulation of acylcarnitines in serum from cocaine-treated mice (30 mg/kg/day, intraperitoneal administrations for three consecutive days) and the inhibition of fatty acid oxidation is known to play an important role in cocaine-induced liver injury [42].

Amino acids are small-molecule metabolites that not only participate in building peptides and proteins but also perform important functions in gene expression in cells, synthesis and secretion of neurotransmitters and hormones, nutrient metabolism, oxidative defense, intracellular protein turnover, immune function, reproduction, obesity, diabetes, and metabolic syndrome [43]. Amino acid profiling has been used for the diagnosis of a variety of diseases such as hepatic fibrosis and cancer in clinical studies [44,45]. Moreover, the levels of neuroactive amino acids, including glutamate, glutamine, gamma-aminobutyric acid, glycine, tryptophan, and D-serine, associated with brain functions, as well as other amino acids were significantly altered in brain diseases such as Alzheimer’s disease in brain specimens [46] and autism spectrum disorders in body fluids (plasma, serum, urine etc.) [47]. Only a few studies on amino acid profiling related to MA exposure have been conducted. In a previous study, dynamic changes were observed in the biosynthesis/metabolism of amino acids, including the phenylalanine, tyrosine, and tryptophan biosynthesis and the valine, leucine, and isoleucine biosynthesis, during 24 h of short-term abstinence in plasma from MA self-administering rats [48]. In another previous study, administration of a single dose (10 mg/kg) or escalating doses (10–30 mg/kg) of MA for five consecutive days in rats was followed by withdrawal for two days, after which 20 amino acids were profiled in the rat serum. Most amino acids including alanine, glycine, and three branched chain amino acids (valine, leucine, and isoleucine) were significantly depleted while levels of glutamate and lysine were elevated with the administration of MA. After two days of withdrawal, most of these levels were restored. The authors suggested that the decrease in branched chain amino acid levels was probably due to elevated energy metabolism, and alterations in levels of other amino acids were related to the activation of the nervous system [10]. On the other hand, in the plasma from MA-induced conditioned place preference model rats (2 mg/kg for 10-day conditioning), no significant changes were observed for most amino acid levels [12]. Our results demonstrate that only two amino acids, arginine and methionine, were dysregulated in the long term. The levels of amino acids in plasma or serum after MA exposure were inconclusive and provided only a snapshot depending on the severity of MA exposure and different abstinence times, whereas changes in amino acids in hair might reflect a more steady change and have the potential for deciding possible biomarkers.

Notably, in the present study, the detected phosphatidylcholines tended to be present at lower levels in the hair of heavy MA abusers than in those of drug-free controls. Alternatively, all detected lysophosphatidylcholines were significantly elevated in heavy MA abusers. Additionally, all three among the seven SM were upregulated, and sphinganine, a precursor of ceramide, which is converted into a variety of sphingolipids, was down-regulated as shown in the results from the untargeted metabolomics. The dysregulation of membrane lipids, such as glycerophospholipids and sphingolipids, due to psychostimulants, such as MA and cocaine, have been previously demonstrated in human and animal studies [48,49,50,51,52]. However, the changes in these studies varied depending on the chemical nature of these lipids, sampling sites such as subparts of the brain [49,50], and sampling time [48]. In particular, the levels of some of lysoPC, PC and SM fluctuated during 24 h of abstinence following MA self-administration in rat plasma [48]. In a previous study, a significant increase in ceramide contents was noted, whereas no difference was observed in the levels of PCs and SMs in the brains of rats following MA self-administration (0.1 mg/kg, FR1, 15-hour sessions for eight days). Thus, the study concluded that MA is involved in cell cycle control and inflammation via the stimulation of the production of the sphingolipid messenger ceramide [53]. Nevertheless, rat blood phospholipid levels were significantly differentiable between cocaine-treated and control groups based on PCA [50]. Furthermore, a reduction of calcium-stimulated phospholipase A2 level was reported in the postmortem putamen tissue of chronic MA or cocaine users [49]. The current study observed elevated levels of lysoPCs and reduced levels of PCs in hair of heavy MA abusers, implying that phospholipase A2 is ultimately activated owing to heavy MA abuse and could serve an important role in MA addiction.

Pathway-based analysis is routinely performed to discover the biological roles of changed metabolites; however, it is not straightforward to divulge the metabolic effects, due to limited numbers of identified metabolites and difficulty in elucidating functional linking to those metabolites. To overcome this challenge, we applied PIUMet to the significantly altered metabolites from the targeted metabolomics as well as ion features from untargeted metabolomics. Our results indicate that lipid metabolism was affected by heavy MA abuse. The perturbation in peroxisome metabolism and biogenesis was detected as shown in Module 1. Peroxisomes are known to play an essential role in ether phospholipid biosynthesis [54]. The genes of galactosyltransferase, sphingomyelin phosphodiesterase, and phospholipase A2 families were detected as critical node genes in Modules 2, 4, 6, and 8 of lipid metabolism. A functional disorder of lipid metabolism is found, conspicuously, in neuropathologies and brain injuries, and the important role of lipids in cell signaling and tissue physiology was proved in numerous neurological disorders [55]. In particular, the activation of phospholipase A2 was clearly demonstrated by the results of the targeted metabolomics.

Metabolic changes in biofluids such as the blood and urine can reflect dynamic changes and facilitate the understanding of mechanistic processes through which pathological or physiological effects develop [56,57]. On the other hand, metabolic changes in the hair could provide insight into important holistic metabolic effects. Therefore, the further investigation of the altered metabolites in the current study for the time of abstinence, possibly to the time of recovering MA addicts, is necessary to discover practical biomarkers.

## 5. Conclusions

The present study demonstrated that hair has great advantages as a diagnostic specimen for determining drug addiction as the endogenous compounds that accumulate in hair are retained. Considering the data from the targeted and untargeted metabolomics, the biosynthesis or metabolism of lipids, such as glycosphingolipids, sphingolipids, glycerophospholipids, and ether lipids, as well as the metabolism of amino acids (glycine, serine and threonine; cysteine and methionine) is affected by heavy MA abuse. In particular, the genes of the key regulating enzymes, including phospholipase A2, for heavy MA abuse are proposed as potential targets for future therapies. These findings highlight the crucial metabolic perturbations caused by MA addiction and will aid in identifying robust diagnostic markers and therapeutic targets.

## Figures and Tables

**Table 1:** Study subjects and hair concentrations of methamphetamine (MA) and amphetamine (AM).

Subjects		ID	Age (Years)	Concentrations (ng/mg)	
				MA ^1^	AM ^2^
Drug-free controls (male, *n* = 12)	Mean (SD)	C1	36	-	-
		C2	34		
		C3	40		
		C4	40		
		C5	42		
		C6	44		
		C7	48		
		C8	35		
		C9	40		
		C10	41		
		C11	40		
		C12	37		
			39.8 (3.9)		
Heavy MA abusers (male, *n* = 10)		M1	37	65.7	4.6
		M2	36	163.4	8.5
		M3	36	72.5	0.2
		M4	27	28.5	1.9
		M5	37	48.7	2.4
		M6	46	31.2	2.7
		M7	30	75.8	6.3
		M8	37	63.74	4.3
		M9	38	25.2	3.7
		M10	46	25.2	2.0
	Mean (SD)		37.0 (5.9)	60.0 (41.5)	3.7 (2.4)

^1^ Methamphetamine; ^2^ Amphetamine.

**Table 2:** Number of metabolites investigated in human hair.

Metabolite Group	Total Number of Metabolites	Number of (Semi-) Quantified Metabolites	Number of Significant Changed Metabolites
Acylcarnitines	40	22	5
Amino acids and biogenic amines	42	22	2
Glycerophospholipids	90	39	25
Sphingolipids	15	7	3
Monosaccarids	1	0	0
Total	188	90	35

**Table 3:** Significantly changed metabolites in the hair samples from heavy MA abusers.

Metabolite Group	Metabolite	Fold Change	*p* Value
Acylcarnitines	Carnitine	1.80	0.0065
	Decadienylcarnitine	2.51	0.0119
	Octadecadienylcarnitine	2.39	0.0224
	Octadecanoylcarnitine	−1.45	0.0047
	Valerylcarnitine	−1.92	0.0062
Amino acids and biogenic amines	Arginine	5.61	0.0044
	Methionine	−1.56	0.0049
Glycerophospholipids	lysoPC a ^1^ C160	2.14	0.0007
	lysoPC a C170	1.73	0.0002
	lysoPC a C181	2.74	4.0469 × 10^−5^
	lysoPC a C204	1.33	0.0478
	PC aa ^2^ C341	−1.52	0.0092
	PC aa C362	−1.41	0.0185
	PC aa C365	−2.78	7.5669 × 10^−5^
	PC aa C366	−2.13	0.0010
	PC aa C381	−1.59	0.0382
	PC aa C383	−1.37	0.0091
	PC aa C385	−2.33	0.0002
	PC aa C403	−2.56	0.0043
	PC aa C404	−2.22	0.0061
	PC ae ^3^ C340	−1.89	0.0033
	PC ae C360	−1.69	0.0015
	PC ae C361	−1.61	0.0054
	PC ae C365	−3.13	0.0001
	PC ae C380	−1.59	0.0020
	PC ae C381	−2.04	0.0026
	PC ae C382	−2.08	0.0035
	PC ae C383	−2.08	0.0078
	PC ae C401	−2.78	0.0005
	PC ae C402	−2.27	0.0071
	PC ae C422	−2.04	0.0150
	PC ae C423	−1.92	0.0284
Sphingolipids	SM ^4^ C241	2.04	0.0184
	SM OH C221	1.77	0.0163
	SM OH C241	1.51	0.0236

^1^ Lysophosphatidylcholine acyl; ^2^ Phosphatidylcholine diacyl; ^3^ Phosphatidylcholine acyl-alkyl; ^4^ Sphingomyelin, SM.

**Table 4:** Metabolites identified in the hair via LC-QTOF-ESI-MS analysis using in-house database.

Ionization Polarity	*m*/*z*	t_R_ (min)	Δt_R_ (min)	Formula	Mass	ΔMass (ppm)	Metabolites (Fold Change)	Species	Score
Positive	130.0507	1.14	−0.31	C_5_H_7_NO_3_	129.0434	−6.49	5-Oxo-proline	(M + H)^+^	83.5
	132.1022	1.32	0.03	C_6_H_13_NO_2_	131.0949	−2.11	(L-)Isoleucine	(M + H)^+^	99.4
	139.0523	1.42	0.00	C_6_H_6_N_2_O_2_	138.0452	−16.54	Urocanate	(M + H)^+^	70.6
	146.0926	1.27	0.14	C_5_H_11_N_3_O_2_	145.0852	−0.41	* 4-Guanidinobutanoate (2.8)	(M + H)^+^	83.6
	195.0873	14.64	−0.04	C_8_H_10_N_4_O_2_	194.0800	1.84	Caffeine	(M + H)^+^	99.1
	302.3051	35.96	0.01	C_18_H_39_NO_2_	301.2978	0.88	* Sphinganine (−1.8)	(M + H)^+^	99.6
Negative	151.0258	2.64	0.01	C_5_H_4_N_4_O_2_	152.0330	2.63	Xanthine	(M − H)^−^	98.1
	167.0207	1.75	−0.07	C_5_H_4_N_4_O_3_	168.0280	1.87	* Urate (−2.0)	(M − H)^−^	86.6
	117.0193	1.51	0.03	C_4_H_6_O_4_	118.0266	0.18	Succinate	(M − H)^−^	87.7
	281.2484	39.56	−0.04	C_18_H_34_O_2_	282.2556	0.92	* Petroselinic acid (−3.3)	(M − H)^−^	99.4
	105.0199	0.90	−0.02	C_3_H_6_O_4_	106.0272	−5.39	Glycerate	(M − H)^−^	85.5
	89.0250	1.04	0.19	C_3_H_6_O_3_	90.0323	−6.46	Glyceraldehyde	(M − H)^−^	85.4
	131.0824	0.85	0.13	C_5_H_12_N_2_O_2_	132.0896	2.13	(L-)Ornithine	(M − H)^−^	85.1
	174.0882	0.85	0.09	C_6_H_13_N_3_O_3_	175.0955	0.97	Citrulline	(M − H)^−^	87.1
	128.0354	1.48	0.03	C_5_H_7_NO_3_	129.0427	−0.49	5-Oxo-proline	(M − H)^−^	87.7
	121.0294	9.68	0.14	C_7_H_6_O_2_	122.0367	0.84	* 4-Hydroxybenzaldehyde (2.9)	(M − H)^−^	87.8

* *p* < 0.05. Fold changes are parenthesized. Underlined metabolites were additionally confirmed using MS/MS analysis.

**Table 5:** Altered metabolism pathways identified in the hair from heavy MA abusers.

Module	Metabolism Pathway	Number of Hidden Proteins	*p*-Value	FDR ^1^	Hidden Protein
1	Peroxisome	4	9.99 × 10^−8^	1.40 × 10^−6^	ACOT8,PEX5,HACL1,PAOX
2	Glycosphingolipid biosynthesis—lacto and neolacto series	3	2.71 × 10^−7^	1.38 × 10^−5^	B3GALT5,B3GNT3,A4GALT
3	Glycine, serine and threonine metabolism	4	5.44 × 10^−9^	1.09 × 10^−7^	SHMT2,SHMT1,GNMT,SARDH
4	Sphingolipid metabolism	3	1.42 × 10^−6^	3.42 × 10^−5^	SMPD3,SMPD2,SMPD4
5	Cysteine and methionine metabolism	3	2.48 × 10^−6^	1.74 × 10^−5^	BHMT2,MTR,BHMT
6	Glycerophospholipid metabolism	4	2.69 × 10^−8^	5.38 × 10^−7^	LYPLA2,LYPLA1,PLA2G2C,PLA2G15
7	Pentose and glucuronate interconversions	3	1.09 × 10^−7^	2.05 × 10^−6^	UGT2B4,GUSB,UGT2B7
	Drug metabolism—other enzymes	3	1.36 × 10^−6^	8.15 × 10^−6^	UGT2B4,GUSB,UGT2B7
8	Ether lipid metabolism	4	2.99 × 10^−10^	4.18 × 10^−9^	PLA2G12B,PLA2G7,PAFAH1B3,PAFAH2

^1^ False discovery rate.

## Supplementary Materials

The following are available online at https://www.mdpi.com/1422-0067/21/17/6041/s1, Supplementary material 1: results of ROC curve analysis, Supplementary material 2: metabolites identified in the hair via LC-QTOF-ESI-MS analysis using PIUMet, Supplementary material 3: list of node genes in the altered metabolic pathways identified using PIUMet.
